# Three Pathways for Standardisation and Ethical Disclosure by Default under the European Union Artificial Intelligence Act

**DOI:** 10.1016/j.clsr.2024.105957

**Published:** 2024-07

**Authors:** Johann Laux, Sandra Wachter, Brent Mittelstadt

**Affiliations:** * Oxford Internet Institute, University of Oxford; ** Oxford Internet Institute, University of Oxford; *** Oxford Internet Institute, University of Oxford

**Keywords:** Artificial Intelligence, AI Act, Standardisation, AI Ethics, Regulation

## Abstract

Under its proposed Artificial Intelligence Act (‘AIA’), the European Union seeks to develop harmonised standards involving abstract normative concepts such transparency, fairness, and accountability. Applying such concepts inevitably requires answering hard normative questions. Considering this challenge, we argue that there are three possible pathways for future standardisation under the AIA. First, European standard-setting organisations (‘SSOs’) could answer hard normative questions themselves. This approach would raise concerns about its democratic legitimacy. Standardisation is a technical discourse and tends to exclude non-expert stakeholders and the public at large. Second, instead of passing their own normative judgments, SSOs could track the normative consensus they find available. By analysing the standard-setting history of one major SSO, we show that such consensus tracking has historically been its pathway of choice. If standardisation under the AIA took the same route, we demonstrate how this would lead to a false sense of safety as the process is not infallible. Consensus tracking would furthermore push the need to solve unavoidable normative problems down the line. Instead of regulators, AI developers and/or users could define what, for example, fairness requires. By the institutional design of its AIA, the European Commission would have essentially kicked the ‘AI Ethics’ can down the road. We thus suggest a third pathway which aims to avoid the pitfalls of the previous two: SSOs should create standards which require “ethical disclosure by default”. These standards will specify minimum technical testing, documentation, and public reporting requirements to shift ethical decision-making to local stakeholders and limit provider discretion in answering hard normative questions in the development of AI products and services. Our proposed pathway is about putting the right information in the hands of the people with the legitimacy to make complex normative decisions at a local, context-sensitive level.

## 1 Introduction

The scientific field of Artificial Intelligence (‘AI’) research dates back to at least the 1950s and yet, the technology is still early in its life cycle.^1^ Successful applications in fields like healthcare or transportation are a rather recent phenomenon. They have been brought about by increases in computational power and access to training data which led to advances in machine learning (‘ML’), a subfield of AI.^2^ Hurdles for a broader implementation of AI in private and public institutions remain. One such barrier is the current lack of standardisation.

Consider a recent example. The COVID-19 pandemic led to great efforts to build AI tools to diagnose the disease and predict patient risks.^3^ The results were underwhelming. Several academic reviews found that none of the AI tools made a real difference.^4^ Some were potentially harmful.^5^ One major challenge was found in the poor quality of the data used by researchers to build the AI.^6^ As one review study finds, researchers drew on public datasets from radiological imaging to train their ML models, but the datasets were either not large enough or not of suitable quality and all models trained on public datasets exhibited a high or unclear risk of bias.^7^ A significant contributor to these difficulties was a lack of proper data standardisation. As the Alan Turing Institute notes: “Different data standards and codification of metadata, and lack of dataset documentation, meant that data were difficult to find, link and assess in terms of missingness and biases, limiting the scope of and confidence in analyses.”^8^

Initiated by market needs, standardisation allows to define voluntary technical or quality specifications for current or future products and services.^9^ It thus offers benefits for research and industry through coordination. Regulators turn to standards, too, when they seek to consolidate expert knowledge to address risks, such as in safety regulation.^10^ They use standards to promote the implementation of legal requirements and ethical values.^11^ Even technical standards are rarely purely technical. They can reflect commercial interests, political preferences, or moral judgments.^12^ This renders standards a powerful regulatory tool. Their importance is visible in the current policy competition for AI standardisation between the world’s economic blocs, especially between the United States, China, and the European Union (‘EU’).^13^

As part of this global race, the European Commission published its proposal for an Artificial Intelligence Act (‘AIA’) in April 2021.^14^ On one hand, the proposal aims to create a functioning European internal market for AI systems and provide legal certainty to facilitate investment and innovation.^15^ On the other hand, the AIA seeks to ensure the safety and trustworthiness of AI systems as well as their conformity with fundamental rights and European values.^16^ This dual purpose of both market making and market regulation is typical for EU regulatory law.^17^ Moreover, EU law can have the power to regulate global markets beyond its jurisdiction through market forces alone (the so-called Brussels Effect).^18^ Some authors predict the AIA to enjoy global impact via the Brussels Effect.^19^

The AIA is based on the EU’s so-called New Approach (today, the New Legislative Framework (‘NLF’)). The New Approach establishes product regulation regimes under which manufacturers assess the conformity of their product with essential requirements specified by law before placing the product on the market.^20^ While manufacturers remain free to interpret the essential requirements themselves, the European Commission can mandate European standard setting organisations (‘SSOs’) to develop harmonised standards which manufacturers may then opt to follow and enjoy a presumption of conformity.^21^ While the European Commission retains its control over drafting EU law, SSOs control the development of technical standards.^22^ The New Approach’s division of labour has been praised as successfully overcoming political obstacles to agreeing upon harmonised European standards, as EU Member States often sought to benefit their domestic industries through standardisation.^23^ Harmonised standards are supposed to aid the implementation of the AIA, rendering standardisation one of the most salient issues around the novel regulation.^24^

Title III AIA contains specific rules for AI systems which create a high risk to the health and safety or fundamental rights of natural persons.^25^ Title III, Chapter 2 AIA then sets out the essential legal requirements for high-risk AI systems. Importantly, and in line with the New Approach, conformity with these requirements will be presumed by adherence to yet-to-be developed harmonised standards on AI (Art. 40 AIA). By following these standards, providers of AI systems do not have to interpret the meaning of the essential requirements in Title III, Chapter 2 AIA themselves while enjoying the presumption of conformity.^26^ In May 2023, the European Commission sent a standardisation request to the European Committee for Standardisation (‘CEN’) and the European Committee for Electrotechnical Standardisation (‘CENELEC’) in support of the AIA.^27^ Both CEN and CENELEC are European SSOs.

There is currently great scholarly and political interest in and speculation about the possible future pathways for the development of AI standards in the EU.^28^ Standardisation as a governance tool has been widely criticized as lacking legitimacy. Largely a technical discourse, it tends to exclude non-expert stakeholders and the public at large.^29^ Industry representatives exert great influence in SSOs.^30^ Standardisation by technical experts can nevertheless be highly political.^31^ At this point in time, it remains an open question to what extent CEN and CENELEC will address hard normative questions which the implementation of AI systems in private and public organisations inevitably raises. Avoiding human fatalities, for example, may be an easily agreeable normative aim. More complex normative issues, such as the acceptability and mitigation of pre-existing biases in training data, will surely be much harder to negotiate between interest groups. Answering “hard normative questions” thus means endorsing specific interpretations or theoretical approaches for normative concepts (e.g., equality, transparency, dignity), or specifying acceptable or preferred trade-offs between competing interests.

Considering these normative challenges, we argue that there are three possible pathways for future standardisation of hard normative questions under the AIA. First, European SSOs could answer such questions themselves (section 2.). This approach would inevitably raise the legitimacy concerns mentioned above. In addition, there is considerable normative uncertainty around the use of AI in society.^32^ This poses a practical problem for SSOs which aim to find specific, coherent, and consensus-based answers for hard normative questions.

Second, instead of passing their own normative judgments, SSOs could track whatever normative consensus they find available (section 3.). SSOs would then defer to norms posited in documents with a higher pedigree of democratic legitimacy than that of standards, such as national and international laws. This carries the risk of SSOs extending the scope of private standardisation towards translating legal principles. Below we are analysing the standard-setting history of one major SSO and show that such consensus tracking has historically been its pathway of choice. If standardisation under the AIA took the same route, we demonstrate how this would lead to a false sense of safety as the process is not infallible. Moreover, we show how normative uncertainty has previously resulted in a standardisation effort being watered down from a document against which conformity can be assessed to a mere guidance document which does not allow for certification. A similar outcome for standardisation under the AIA would be incompatible with the European Commission’s explicit goal of developing certifiable standards (Art. 44 AIA), including CE marking for high-risk AI systems (Art. 49 AIA). Consensus tracking would furthermore push the need to solve unavoidable normative problems down the line. Instead of regulators, AI developers and/or users could define what, for example, fairness requires within a concrete implementation of AI. By the institutional design of its AIA, the European Commission would have essentially kicked the ‘AI Ethics’ can down the road.^33^

Third, we argue that this would be a missed opportunity to address hard normative questions in AI standardisation (section 4). We thus suggest a third pathway which aims to avoid the pitfalls of the previous two: SSOs should create standards which require “ethical disclosure by default.” These standards will specify minimum technical testing, documentation, and public reporting requirements to shift ethical decision-making to local stakeholders and limit provider discretion in answering hard normative questions in the development of AI products and services. Rather than setting specific ethical requirements for trade-offs and thresholds, this approach would instead ensure that all providers of AI systems meet a minimum harmonised standard for testing, reporting, and public participation. Ethical disclosure by default would exceed the reporting and participatory obligations currently proposed in the AIA draft regulation. Compliance would require AI providers to furnish relevant third parties with a standardised set of test results and documentation to enable local decision-makers to set normative requirements in a procedurally consistent way. Our proposed pathway is about putting the right information in the hands of the people with the legitimacy to make complex normative decisions at a local, context-sensitive level.

## 2 Pathway One: Standardisation Bodies Answering Hard Normative Questions Themselves

As mentioned, the legitimacy of standardisation is an ongoing concern in the literature.^34^ The delegation of regulatory authority to SSOs raises issues of civil society and non-expert participation in the production of standards (input legitimacy),^35^ representation of public policy interests in the standards developed (output legitimacy), and accountability and transparency of the institutional decision-making processes as well as in the implementation of standards (throughput legitimacy).^36^ It is to be expected that the development of standards under the AIA will be extremely value-laden and expose competing, if not incommensurable interests.

It would thus be surprising if SSOs under an AIA mandate would aim to provide solutions to hard normative questions on sensitive topics such as accuracy or performance thresholds, equal distribution of outcomes between protected groups, acceptability of biases, degrees of interpretability and transparency, and monitoring of emergent risks after an AI system has been placed on the market, to name just a few. As mentioned, answering “hard normative questions” means endorsing specific interpretations or theoretical approaches for normative concepts (e.g., equality, transparency, dignity), or specifying acceptable or preferred trade-offs between competing interests. In contrast, standards with “high level ethical content” will provide overviews of issues such as relevant concepts, interests, and stakeholders, potential interpretations, or theoretical approaches for understanding them, or methods for “embedding” them in a system through design choices, testing, auditing, or management.

Moreover, it is unclear whether answers to such questions could even be provided at the level of a technical or regulatory standard. Determining an equal distribution of outcomes or performance in a classification task, for example, requires knowledge of the specific application, production environment, group composition, and classes or resources to be distributed, among other considerations. Standards tend to address a higher level of abstraction than such case-specific decisions.

The EU has its own Regulation on European Standardisation which defines the “primary objective” of standards in the EU to lie in the “the definition of voluntary technical or quality specifications with which current or future products, production processes or services may comply.”^37^ Answering questions of legality beyond providing a presumption of conformity with harmonised standards is thus not within the remit of SSOs in the EU.^38^

If SSOs nevertheless opted to draft AI standards which address such hard normative questions, they would have to satisfy several demands of legitimacy. At a minimum, they would have to consult a large set of diverse bodies and expert groups (input legitimacy),^39^ keep the standardisation process transparent and accountable (throughput legitimacy), and make sure that the adopted standards are responsive to the interests of all affected groups (output legitimacy).

The so far short history of AI regulation in the EU shows how difficult it can be for mixed-interest decision-making bodies to arrive at specific rules. The 52-member High-Level Expert Group on Artificial Intelligence (‘HLEG AI’) worked on Ethics Guidelines for Trustworthy AI for nine months but could not agree on “red lines” for AI: “non-negotiable” ethical principles which prohibit specific uses for AI in the EU. Instead, the adopted text merely mentions “critical concerns” for certain AI uses.^40^

A more practical problem for SSOs lies in the state of consensus about AI’s normative implications. Take fairness as an example. What it demands of decision-makers in society is both the subject of ongoing academic research as well as continuous judicial decision-making, for example under non-discrimination law.^41^ Meanwhile, a budding new field of literature on “algorithmic fairness” has provided an inconsistent set of quantitative definitions of fairness for AI-based decisions.^42^ The aim of quantifying and thus automating fairness is, however, misguided. While consistent standards of fairness metrics can indeed provide important assessment procedures for automated systems, they cannot dispense with the need for contextual assessments by local decision-makers such as judges.^43^

More generally, there are (at least) four key challenges for translating abstract ethical principles into concrete and verifiable requirements for AI development. First, there is a lack of common aims and fiduciary duties in AI development, unlike in formal professions with aims that align with public interests such as in medicine. Second, AI developers cannot draw on a long-standing professional history and established norms of ethical behaviour. Third, there a lack of proven methods for effectively translating high-level ethical principles into practical guidelines and actions in AI. Fourth and finally, legal and professional accountability and redress mechanisms are still insufficient in AI.^44^

There is thus a considerable amount of normative uncertainty around the use of AI in society. What the law and ethics demand in a specific use case of an AI system cannot always be known in advance. It likewise may be subject to principled disagreement over the correct meaning of essentially contested concepts (e.g., fairness, dignity) and thus not resolvable by consensus.^45^ Considering these challenges, it seems unlikely that SSOs will directly answer hard normative questions themselves. Where available, they will likely aim to track existing normative consensus, as we show in the following section.

## 3 Pathway Two: Standardisation Bodies Tracking Normative Consensus

The second pathway leads SSOs to track existing normative consensus as far as it is available, meaning they will defer to norms posited in documents with a higher pedigree of democratic legitimacy than that of standards, such as national and international laws. Below we show that consensus tracking has indeed been the avenue of choice for SSOs. While previous European SSOs have produced standards including high-level ethical content such as the “Guidance for the Responsible Development of Nanotechnologies” (CEN/TC 16937:2016), the “European Professional Ethics Framework for the ICT Profession” (CEN/TS 17834), and the “Ethics Assessment for Research and Innovation” (CWA 17145-1:2017; CWA 17145-2:2017),^46^ CEN and CENELEC are still at an early stage of developing standards for AI. However, there is more mature standardisation work on AI by the International Organization for Standardization (‘ISO’), a worldwide federation of national standards bodies.^47^

European SSOs mandated under the AIA will be able to draw on existing standards and technical specifications through cooperation agreements such as the so-called Vienna agreement between CEN and ISO or the so-called Frankfurt agreement between CENELEF and IEC.^48^

By drawing on two case studies, ISO 26000 (section 3.1) and the work of ISO subcommittee 42 (section 3.2), we show the hesitancy of SSOs to answer hard normative questions beyond the tracking of available normative consensus and abstract and vague references to assessment tools and metrics. Trends identified through this analysis suggest that future standardisation work on AI by CEN and CENELEC (section 3.3) will unlikely produce detailed, specific, and coherent normative thresholds and requirements for the development and use of AI in the EU. We lay out below why this can have troubling consequences.

### 3.1 Example 1: ISO 26000

The genesis of ISO 26000 provides a good example of consensus tracking in standardisation and its challenges. The standard aims to provide guidance on social responsibility, but is widely regarded as a missed opportunity.^49^ For ISO, it was an “out-of-the-box” project: ISO had so far been known for developing technical standards and not for guiding public policy issues such as social responsibility.^50^ ISO’s work is normally carried out through technical committees to which each interested ISO member body has a right to be represented in.^51^

As Bijlmakers and van Calster reconstruct the origin of ISO 26000, the standard was first intended to become a specification document – a so-called “management systems standard” (‘MSS’) – which would have allowed certification and conformity assessments.^52^ Such was the recommendation of ISO’s Committee on Consumer Policy (‘COPOLCO’), a group comprised of member standardisation bodies and thus mainly reflecting the interests of industry.^53^ ISO, however, also installed the Strategic Advisory Group on Social Responsibility (‘SAG’), which represented more diverse interests of business, labour, environmental groups, consumer groups, civil society, United Nations bodies and developing countries.^54^ The SAG suggested that ISO 26000 should only be a “guidance document” and not a “specification document against which conformity can be assessed.”^55^ ISO, the SAG recommended, should only proceed with standard ISO 26000 if it recognises “that social responsibility involves a number of subjects and issues that are qualitatively different from the subjects and issues that have been already dealt with by ISO.”^56^ The SAG further recommended that ISO “narrows the scope of the subject so as to avoid addressing issues that can only be resolved through political processes.”^57^

The SAG recommendations had significant influence on the genesis of ISO 26000.^58^ As ISO states, ISO 26000 “does not contain requirements and, as such, cannot be used for certification. Any offer to certify, or claims to be certified, against ISO 26000 would be a misrepresentation of its intent and purpose.”^59^ It appears that the recommendation to develop a mere guidance document was based on the desire to find consensus among the competing interests represented in the SAG.^60^ As Bijlmakers and van Calster note, finding consensus for a certifiable standard would have proven to be difficult. At the time of creating ISO 26000, “[t]he concept of [social responsibility] and its substantive issues had not sufficiently matured yet.”^61^ In retrospect, Hahn argues that ISO’s insistence that ISO 26000 is not an MSS and thus not certifiable “was at least partly made to avoid a misuse of ISO 26000 as a facade pretending responsible business conduct.”^62^

The SAG further emphasised both substantive and procedural matters of legitimacy.^63^ The SAG problematised ISO’s lack of legitimacy to define substantive elements of social responsibility. ISO, it stated, “does not have the authority or legitimacy to set social obligations or expectations which are properly defined by governments and intergovernmental organizations.”^64^ This meant that ISO should defer to relevant standards not least set by international law with its higher degree of democratic legitimacy.^65^ In effect, ISO 26000 should thus track existing normative consensus. The organisational pedigree of such consensus was also considered, as similar standardisation efforts were taking place at that time. The SAG recommended that ISO recognises “the difference between on the one hand, instruments adopted by authoritative global inter-governmental organizations (such as the United Nations Universal Declaration on Human Rights, international labour conventions and other instruments adopted by the ILO and relevant UN Conventions) and on the other hand, private voluntary initiatives that may or may not reflect the universal principles contained in the above instruments.”^66^ The SAG thus preferred public mandates over private initiatives.^67^ As regards ISO’s procedural legitimacy, emphasis was placed on the need for appropriate stakeholder involvement.^68^

The genesis of ISO 26000 also shows that consensus tracking can misfire. Members of ISO’s International Working Group on Social Responsibility (‘WGSR’) voiced their concerns about the standard’s use of the ‘precautionary principle’ and its effects on states’ obligations in terms of (customary) international law.^69^ There was significant debate about the inclusion of the precautionary principle in ISO 26000 and whether or not (and if so, to what degree) the principle should be phrased as being sensitive to cost-effectiveness.^70^ There was no consensus amongst interested states on this issue. Some governments thus worried that the behaviour of governmental experts in the drafting procedure may be understood as signalling state practice. This could have contributed to the creation of customary international law, adopting a particular understanding of the precautionary principle. Though formally acting as ‘independent experts’, delegates to the WGSR thus started responding to the political positions and legal interests of their respective governments.^71^ In the end, the agreed-upon text of the standard did, to a large degree, not go beyond well-accepted principles of international environmental and sustainable development law.^72^ Thus, only after political intervention was the tracking of (a minimum) consensus successful.

The drafting history of ISO 26000 shows both the appeal for SSOs to track normative consensus and the intricacies of doing so. Consensus may not be available beyond a minimum degree of abstract principles. Moreover, the attempt to track consensus correctly may fail.

### 3.2 Example 2: The Work of ISO SC 42

In 2017, ISO launched the development of standards on AI. Its subcommittee 42 (‘SC 42’) works to “create an ethical AI-enabled society.”^73^ SC 42 was set out to develop an MSS, i.e., the type of standard which ISO 26000 was initially thought to become and was then watered down to a guidance document. The MSS for AI is supposed to “establish specific controls, audit schemes and guidance that are consistent with emerging laws, regulations and stakeholder needs.”^74^ ISO advertises MSSs as providing “common building blocks, and risk management frameworks, for companies, governments, and other organizations.”^75^ As regards AI, the MSS approach is supposed to “[e]nable organizations to dynamically map their work to the regulatory and societal requirements captured through the MSS; [b]e a trust mechanism that will facilitate B2B contracting, [e]stablish a baseline that can be verified through audit and/or conformity assessment.”^76^Against the backdrop of the history of ISO 26000, it is noteworthy that SC 42 does not shy away from invoking normative concepts. Its approach seeks to “accelerate AI adoption whilst simultaneously addressing fairness, accountability and ethical concerns.”^77^ In December 2023, ISO published ISO/IEC 42001, the world’s first MSS for AI.^78^ The standard addresses, amongst other issues, ethical considerations. It lists specific issues that should be considered in an organisation’s design of management processes related to AI, including “concerns related to the trustworthiness of AI systems such as security, safety, fairness, transparency, data quality and quality of AI systems throughout their life cycle”.^79^

Note, however, that not all ISO MSS are certifiable. There are at least three different types of MSS and only the most elaborate and strict type of an “MS requirements standard” foresees certification.^80^ As of February 2024, SC 42 has published 25 standards, including so-called technical reports and technical specifications.^81^ Below, we are analysing four of these 17 documents in more detail. We begin with three technical reports. Our overall assessment is that they first and foremost lay out technical mitigation strategies. This is somewhat expected for technical reports of an SSO. However, their explicit dealing with normative concerns remains largely cursory.

#### 3.2.1 ISO/IEC TR 24028

The first of the three technical reports analysed here is ISO/IEC TR 24028 (“Overview of trustworthiness in artificial intelligence”).^82^ The document “surveys topics related to trustworthiness in AI systems” but does not specify levels of trustworthiness.^83^ It speaks to its technical focus that TR 24028 holds trustworthiness to be improvable through “an organizational process with specific measurable outcomes and key performance indicators (‘KPIs’).^84^ The report mentions the precautionary principle as a “risk mitigation technique against potential unintended consequences, such as harm to rights and freedom of natural persons, life of any kind, the environment, a species or a community.”^85^ The history of ISO 26000 outlined above, however, shows how difficult it can be for a standard to spell out the precise contents of the precautionary principle. TR 24028 does not go further in normative consensus tracking either. It does not feature normative red lines for the use of AI or attach concrete values to particular normative interests. Elsewhere, we have argued that trustworthiness is an inherently normative concept which should not be conflated with acceptability benchmarks for technological risks.^86^

TR 24028 does, however, contain what could be called ‘soft’ normative thresholds. For example, it presents the data minimisation principle as a mitigation strategy for privacy threats. While the document acknowledges that collecting less data could protect privacy, it also states that limiting data acquisition can be “challenging” for machine learning models which are dependent on large amounts of data.^87^ Another example of a soft threshold can be found in the comparison of an AI system to human intelligence capabilities: an AI system automating human activity should not perform worse than humans.^88^ This obviously requires determination of threshold values for human performance, requiring “representative data samples.”^89^ Often, however, an AI system’s outperformance of humans at a given task will often be the very reason for its introduction. This benchmark will thus regularly not reach far into the life cycle of an AI system. Moreover, the technical report does not specify the relevant variables of outperformance. AI systems may, for example, outperform humans in speed, accuracy, or reliability of decision-making. These thresholds are thus soft in the sense that they will have to be further specified before being implemented by AI providers. In absence of specification through regulators, it will be left for AI developers or users to fill out their content.

TR 24028 repeatedly refers to ethical values and legal norms. These mentions are, however, of a very broad and general nature. There is a noticeable effort to be inclusive towards various ethical systems. The document states that “different views of trustworthy AI can also result from different moral worldviews or systems of values.”^90^ It continues to state that the “relevance and impact of different worldviews, such as Western Ethics, Buddhism, Ubuntu, Shinto, on AI is still relatively unexplored.”^91^ This essentially leaves possible ethical conflicts unresolved where normative consensus may not be easily available. When addressing privacy as a mitigation measure, TR 24028 leaves it to stakeholders to uphold their values derived from “a particular stakeholder’s ethical worldviews.”^92^ TR 24028 thus continues ISO’s approach of consensus tracking.

#### 3.2.2 ISO/IEC TR 24027

One of the first deliverables of SC 42 has been a “technical report” on bias in AI systems and AI-aided decision making, published in November 2021.^93^ The report lists “current best practices to detect and treat bias.”^94^ The scope of the report lies entirely in describing “measurement techniques and methods for assessing bias […] with the aim to address and treat bias-related vulnerabilities.”^95^

The report visibly aims at a careful and distinguished description of both concepts of bias and fairness.^96^ While bias appears to be the more technical, hence easier-to-define concept, the inherently normative notion of fairness poses a bigger challenge. The report abstains from defining fairness, citing its “highly socially and ethically contextual nature.”^97^ This raises doubts as to the expected substantive normative content of future ISO standards on AI. The technical report constrains itself to describing sources of unwanted bias in AI systems (Clause 6), fairness metrics derived from the literature on algorithmic fairness (Clause 7), and the treatment of unwanted bias throughout an AI system’s life cycle (Clause 8).^98^

In sum, the technical report describes appropriate methods for identifying and reducing bias. It does not, however, define normative thresholds. Instead, these will have to be derived from positive laws and selected moral values. As the report states, the “identification of external requirements” is part of a system’s development and life cycle.^99^ “Special consideration” may be given to the following regulatory requirements: international human rights, equality and indigenous rights instruments; specific laws and guidance relating to the provision of technical solutions (for example accessibility regulations such as the United States Health Insurance Portability and Accountability Act); data protection and privacy legislation (such as the European Union’s General Data Protection Regulation); competition and business law.^100^

Diverging norms and interpretations of national laws and regulations, however, will not provide grounds for a “global consensus”^101^ which can be easily standardised. Tracking consensus by reference to positive laws is, as we have seen with ISO 26000, a challenging task. Moreover, regulation regarding AI and its applications is only beginning to be drafted. There is considerable normative uncertainty around AI. If standardisation is to be “consistent with emerging laws, regulations and stakeholder needs,”^102^ then standardisation must proceed with significant gaps in knowledge. There thus remains the risk that in absence of such regulatory norms, AI developers and users will themselves define thresholds they find normatively acceptable in light of their commercial and organisational interests.

#### 3.2.3 ISO/IEC TR 24368

The third technical report we analysed is TR 24368 (“Overview of ethical and societal concerns”), published in August 2022.^103^ The document finds that “[a]ddressing ethical and societal concerns has not kept pace with the rapid evolution of AI.”^104^ TR 24368 follows the same limited scope of the previously analysed technical reports. It “provides a high-level overview of AI ethical and societal concerns,” but does not “advocate for any specific set of values (value systems).”^105^ TR 24368 references several ethical frameworks, namely virtue ethics, utilitarianism, and deontology, which “can be considered.”^106^

TR 24368 considers ISO 26000 a “fundamental source” for addressing ethical and societal concerns: ISO 26000 “describes social responsibility in a form that can inform activities related to standardising trustworthy AI.”^107^

As did the previous technical reports, TR 24368 makes references to legal frameworks. The document draws particularly on international human rights and international law, such as the Universal Declaration of Human Rights or the International Convention on the Elimination of All Forms of Racial Discrimination.^108^ While no doubt relevant, the approach appears somewhat misbalanced. First, TR 24368 states that international human rights are “fundamental moral principles” to which humans are entitled.^109^ From a legal perspective, however, human rights are to a large degree justiciable legal rights and not “merely” moral principles. Second, the document lists several examples of potential impacts of AI on human rights, all of which are civil and political human rights, such as the right to life or the right to expression.^110^ Now, the document does not touch on important discussions in the legal literature such as positive obligations for states (or businesses) under international human rights law or the justiciability of socio-economic human rights. As an exercise of consensus tracking, these references to international (human rights) law thus risk running into similar problems as ISO 26000 did and a false consensus may be identified.

This finding contrasts with the “AI principles” suggested in TR 24368. These principles are supposed to “support organizations beyond nonmaleficence and to focus on beneficence of technology.”^111^ Instead of simply avoiding harm, AI should be designed with the intention to promote social good.^112^ While clearly context-dependent, it would be a stretch to assume that there is anything available which resembles a global consensus on the social good. For example, one principle listed is the “promotion of human values”, aiming to “ensure that AI is deployed and utilized in a way that maximizes benefit to society, promote humanity’s wellbeing and encourage human flourishing.”^113^ The document then states that “[t]here is literature indicating that some human values are potentially universal.”^114^ This is a rather elusive statement.

#### 3.2.4 ISO/IEC 38507

ISO/IEC 38507 (“Governance implications of the use of artificial intelligence by organizations”), published in April 2022 serves as a guidance document for organisations using AI.^115^ It states that the introduction of AI can create “new obligations” for an organisation. These can be (new) legal requirements or may arise from the adoption of voluntary codes of conduct.^116^ ISO/IEC 38507 mentions that there can be “constraints on the use of AI.”^117^ Annex A sheds some light on what those are. As regards the governance of data use, the standard states that data use is usually constrained: “Some of these [constraints] are imposed externally on the organization through legislation, regulation or contractual obligations and include issues of, e.g. privacy, copyright, commercial interests. Other considerations include ethical or societal obligations, or organizational policies that restrict the use of the data.”^118^ What those legal, ethical, and societal obligations for data use are, remains unspecified. Again, if anything, soft normative thresholds are established. For example, the document states that while decisions such as refusing a bank loan to a customer can negatively impact stakeholders, the use of AI should “not exacerbate” the negative impact.^119^ As with the documents analysed above, reference to ISO 26000 is made.^120^ It is normatively redundant to mention that laws apply to the utilisation of data, as an organisation’s general obligation to comply with the law obviously exists independently from the guidance document. The general purpose of standards is to define quality requirements for products, not to interpret the law. With AI governance, SSOs might, however, see an opportunity to extend the scope of private standardisation towards translating legal principles. As we have seen with ISO 26000 above, this carries the risk of failing to correctly identify the legal status quo.

#### 3.2.5 ISO/IEC 42001

Finally, as mentioned above, ISO/IEC 42001 (“Information technology – Artificial Intelligence – Management system”) is an MSS, published in December 2023. The standard follows a risk-based approach,^121^ which aligns it with the risk-based approach of the AIA. As a full MSS, ISO/IEC 42001 allows certification. To get certified, an organisation must undergo assessment by an external certification body.^122^

ISO/IEC 42001 describes policies and processes for the responsible use of AI systems in organisations.^123^ The document, however, explicitly “avoids specific guidance on management processes.”^124^ Instead, it provides a procedural framework for managing AI systems. This framework is clearly sensitive towards ethical concerns. Issues such as fairness, explainability, and environmental impact are repeatedly mentioned.^125^ The document requires organisations to define, implement and execute several management tools. Prominent amongst these are the formulation of “AI policies” and “AI objectives”;^126^ the conduct of AI risk assessments and risk treatments;^127^ as well as “meaningful” human oversight of AI.^128^

ISO/IEC 42001 neither answers hard normative questions nor does it track normative consensus. It merely states the relevance of ethical and societal/ecological considerations as well as applicable laws (including contractual obligations) and human rights.^129^ It leaves it to organisations to make ethical value choices. Take data quality as an example. The document states that “[t]he organization shall define and document requirements for data quality […]”^130^ It will thus be for organisations themselves to decide whether a certain data set is of sufficient quality for training or testing an AI system. While the document expresses concern for the inclusiveness of data towards specific demographics and potential biases contained in a given data set,^131^ it does not set any specific benchmarks. ISO/IEC 42001 exclusively focuses on prescribing management processes, not on making normative choices. As regards data quality, it requires documentation of the provenance of data, the process for labelling data, and known or potential biases in the data, amongst other issues.^132^ Requiring such documentation is not trivial for AI ethics. Current practice in AI development often omits publishing the instructions for labelling data, albeit their huge influence on the accuracy of a data set.^133^ We will expand on the importance of such information disclosure in the section 4. Lastly, it must be mentioned that at the time of writing, SC42 is developing standards for data quality in machine learning in the ISO/IEC 5259 series.^134^ Whether or not these standards will answer hard normative questions is impossible to know in advance, but unlikely based on our analysis of ISO’s standardisation practice.

### 3.3 Outlook on European Standardisation under the AIA

Based on our analysis of ISO’s work we will now formulate our expectations for future standardisation under the AIA. We will consider three areas of concern.

To begin with, the normative environment of AI is marked by uncertainty. What algorithmic fairness affords, for example, is subject to continuous debate. Arguably, the situation is somewhat comparable to that of ISO 26000, in which development of the concept of ‘social responsibility’ had not sufficiently matured and yet simultaneous standardisation efforts were underway. However, a similar outcome for standardisation under the AIA – i.e., mere guidelines rather than certifiable standards – would be incompatible with the European Commission’s goal of having compliance with the EU’s future AI standards to signal compliance with the AIA (Art. 40 AIA). Certification under AI standardisation is explicitly wanted (Art. 44 AIA), including CE marking for high-risk AI systems (Art. 49 AIA).

What types of standards will be developed under the AIA is thus crucial. SSOs offer a broad range of types and sub-types of deliverables. ISO and CEN/CENELEC each offer full standards, technical specifications, technical reports, and guides, amongst other documents.^135^ Standards can further be differentiated into sub-types such as input or output standards, process standards, or management systems standards (i.e., the MSS mentioned above).^136^ All different types and sub-types fulfil different functions. Only certain (sub-)types of standards are certifiable by third parties. Considering the typology of standards is thus crucial in debates about AI governance.

While CEN’s European Standards allow certification, Technical Specifications provide a normative document where a European Standard is not (yet) feasible, but market guidance is desirable, not least in situations where technologies are still evolving.^137^ Both CEN’s European Standards and Technical Specifications are normative in nature, whereas Technical Reports and Guides are merely informational.^138^ It is thus conceivable that fully certifiable European Standards will only emerge further down the road of the standardisation process. Technical Specifications functioning as “pre-standards”^139^ may have to pave the way first.

As mentioned in section 2, there are good normative and practical reasons for why European AI standards are not going to resolve hard normative questions. This carries the risk, however, that AI certification will give the appearance of safety and consensus where in fact significant normative uncertainty remains. When ISO’s SC 42 states that its standardisation will be consistent with “emerging laws” (see above), this should be understood as a consensus tracking effort which must either provide vague and abstract standards or obfuscate existing uncertainty or principled disagreement.^140^ After all, judicial interpretation of future laws cannot be known in advance. This is especially true where legally protected interests will have to be weighed against each other, as in legal proportionality analyses. This issue likewise persists for European standardisation under the AIA. That said, even though standards developed by ISO and by CEN/CENELEC are comparable as regards their typology and certifiability, only the latter SSOs are mandated with issuing harmonised standards under the AIA, offering the above-mentioned ‘presumption of conformity’ to manufacturers. A potentially influential role in managing the normative uncertainty around AI will be filled by the European Commission’s AI Office. The yet-to-be created AI Office will be tasked with developing and coordinating AI policy at the EU Level and with supervising the implementation and enforcement of the AIA.^141^ It could leverage its role and help CEN/CENELEC determine which normative decisions are appropriate to include in standards.^142^

Second, as the drafting of ISO 26000 and its deference to norms of international law shows, even where some normative consensus is available, its tracking by SSOs can misfire. The standard almost identified consensus wrongly. Deference to law can require significant normative expertise in the ranks of the drafting body. This raises an important institutional question around standard setting: how should SSOs comprised of technical experts best fulfil their mandate to track normative consensus, if at all?

If an SSO aims to demonstrate deference towards non-discrimination law or ethical norms of algorithmic fairness, then this not only raises procedural questions of stakeholder involvement. The institutional dimension of legitimacy prompts us to ask whether this SSO is, relatively speaking, good at tracking consensus compared to other institutions, or more prone to errors or oversimplification.^143^ The composition of an SSO can thus have substantive implications as well. For normative questions such as those posed by the demands of fairness and non-discrimination and to prevent tracking errors, a diverse composition of an SSO in terms of their skillset and domain expertise could be necessary. Alternatively, a re-allocation of decision-making authority could be recommendable. In essence, this means that normatively speaking, ‘merely’ tracking consensus is not a controversy-free strategy for European SSOs acting under an AIA mandate either.

Building on our analysis above, we differentiate three ways in which tracking consensus can fail. First, the search procedure might be faulty. It could, for example, be biased, look at the wrong or incomplete sources, or fail to place equal weight on considerations with equal significance. As much as consensus is supposed to be identified in normative documents, be it laws or ethics frameworks, their (democratic) legitimacy should be taken into consideration, too. This may include the composition of the decision-making group in terms of expertise and representation. It may also require checking whether existing normative ambiguity and principled disagreement is duly acknowledged and not simplify papered over or ignored in favour of conceptual clarity.

Second, consensus tracking may be procedurally sound, but still err in its substantive outcome. As seen above with ISO 26000, there is a risk that although an SSO has identified the correct source for normative content (i.e., international law), its interpretation of the source material could be faulty or based on a misunderstanding (i.e., the correct contours of the precautionary principle in international law).

Third, reluctance on behalf of SSOs to address sensitive issues and provide specific rules for AI means that hard normative questions will have to be answered by local decision-makers. At some point in designing or deploying AI such questions must be answered. AI providers, developers, users, and other stakeholders will thus have to define what, for example, ‘fairness’ means in concrete AI applications. In effect, when an AI system fails to meet the demands of fairness, there is a risk that blame will be shifted to the local human operators and away from institutional decision-makers, i.e., standard setters and AI developers.^144^ Frontline human AI users will regularly have little influence on the system design and political goals behind an AI system.^145^ Yet they are prone to be blamed when AI fails. In sum, the European Commission’s chosen approach to normatively hedge AI may have kicked the can so far down the road that local AI users will have to answer the hardest normative questions and potentially face the biggest backlash.

Based on the genesis of ISO 26000 and SC42’s current work on AI standards, it appears that SSOs lack the institutional design and capacity to reliably address normative issues well. While SSOs might implement institutional redesigns in the future, we will now present an alternative solution, our approach to standardisation called “ethical disclosure by default.”

## 4 Pathway Three: Standardisation Bodies Create Minimum Ethical Disclosure By Default Standards

The recent history and rapid growth in interest in AI ethics and regulation is instructive of the difficulties standardisation processes will face in the near future. Hundreds of frameworks have been published that describe how to make AI ethical and trustworthy. Few offer answers to hard normative questions, preferring instead to seek consensus around a set of common ethical principles, values, or concepts that should somehow be embedded in the development, usage, or regulation of AI. Consensus seemingly exists that thresholds, trade-offs, and specific requirements should be set for ethical AI, and yet reluctance to provide specific, implementable answers to such questions is equally apparent.^146^

In one sense the difficulty encountered in addressing the ethical trade-offs in AI is encouraging, as it reflects the complexity and difficulty of ethics on the ground. Case-specific ethical questions can rarely be answered in a straightforward or top-down manner, instead requiring careful navigation of an array of competing interests, values, and requirements. SSOs are only the latest in a long line of actors expected to make such decisions in pursuit of responsible AI regulation and usage. As explored above, SSOs lack the political legitimacy to answer these questions, and should resist doing so where clear normative consensus cannot be tracked despite the European Commission having seemingly empowered them to provide answers. Consensus tracking alone is unlikely to deliver specific ethical requirements, as universal consensus on what is a ‘good’ life or the ethically ‘correct’ action in a given circumstance is incredibly rare in philosophical and applied ethics. Simply put, technical standardisation processes of the type required under the AIA are ill-equipped to provide specific, implementable answers to hard normative questions.^147^

At the same time, conformity with harmonised standards under the AIA will be presumed to indicate compliance with the requirements of the regulation. It follows that if SSOs fail to provide specific, implementable answers to hard normative questions in these standards, AI providers can be considered compliant while simultaneously setting subjective ethical trade-offs and thresholds without direct external stakeholder involvement. Leaving these questions for AI providers to answer and determine appropriate ethical trade-offs and thresholds for specific applications or cases is equally problematic on legitimacy grounds.

Given the legitimacy concerns already discussed, this outcome of AIA standardisation should be avoided. This does not mean, however, that SSOs should venture into setting specific ethical requirements themselves. Such questions normally fall within the remit of governments, intergovernmental organizations, policymakers, the judiciary, and relevant civil society actors, and should not be left by default to entities or procedures lacking political legitimacy.^148^

Rather than trying in vain to standardise ethical requirements for AI on the ground, our recommended third pathway is for SSOs to create standards which require “ethical disclosure by default.” According to this approach, standards addressing ethical or normative questions should describe minimum testing, documentation, and public reporting requirements. Ethical disclosure by default will shift setting of acceptable ethical trade-offs and thresholds away from AI providers and towards local decision-makers with the legitimacy and knowledge to make such determinations. In other words, ethically relevant AIA standards should intentionally limit AI provider’s discretion in answering hard normative questions, and instead require providers to deliver a consistent minimum evidence base to support local ethical decision-making.

Precise evidentiary requirements will vary between standards. Following the technical focus of CEN/CENELEC standards they should include a range of technical tests and organisational documentation measures available to AI providers. As it is difficult to predict which tests, documentation, and formats will be most useful to specific stakeholders in specific use cases,^149^ the required testing and documentation regime should be tailored to individual standards but appropriately broad and diverse in coverage. Relevant tools may include (for example):

- Bias tests and de-biasing methods, including pre-, in-, and post-processing methods;^150^
- Fairness measures and enforcement methods including individual, group, unawareness, and counterfactual measures, as well as open-source toolkits;^151^
- Transparency and explainability methods including local and global model and outcome explanations, model inspection methods, interpretable models, post hoc explanations;^152^
- Model and data standardised documentation such as datasheets for datasets, model cards, nutrition labels;^153^
- Impact assessments such privacy impact assessments, Algorithmic Impact Assessments, equality impact assessments;^154^
- Any other documentation describing ethical decisions made by providers or procedures used to make such decisions such as internal or external ethics review committees, content moderation policies, model selection criteria, relevant elements of design specifications.

Standards should set minimum requirements for implementation, testing, and documentation using these and other tools in order to establish a common, broad range of tests that provide essential information relevant to local stakeholders responsible for setting case-specific ethical requirements, such as organisations implementing an AI system or competent national authorities overseeing and coordinating AI supervision.^155^

To enact the shift from provider to local ethical determinations, AIA standards should adopt default public disclosure requirements which exceed the current reporting and participatory requirements of the AIA.^156^ Local stakeholders should be empowered to request test results, impact assessments, transparency disclosures and explanations, and any other relevant required documentation. Relevant local stakeholders should include organisations using AI provided by a third party, individuals subject to its use or otherwise affected by it, civil society organisations acting on behalf of such individuals, lawyers and the judiciary, as well as regulators, governmental, and intergovernmental organisations. Eligible stakeholder types, as with the requirements themselves, should be determined on a per standard basis.

Our proposed approach follows the principle of consensus-based standardisation generally adopted by SSOs internationally.^157^ It extends stakeholder participation from setting the standards themselves to also participating actively in their interpretation and enforcement of normative requirements on a case-by-case basis. It likewise aims to mirror and inform the politically legitimate procedures through which such questions of ethics, law, or fundamental rights are normally answered, for example through regulation or judicial decision-making. While perhaps radical in the context of technical standardisation, our approach is modelled on existing procedures for deciding ethical dilemmas in hospitals, universities, courts, and other institutions.^158^

Our approach likewise builds on prior research examining how thresholds and trade-offs are determined in interpreting equality and non-discrimination law in the EU. Specific, measurable, and generalisable thresholds and trade-offs are rarely offered in equality law and jurisprudence. Instead, equality is given meaning on a case-by-case basis.^159^ In seeking to align technical work on fairness in AI with judicial interpretation procedures under equality law, Wachter et al. explained that “establishing a standard set of statistical evidence for automated discrimination cases can help ensure consistent procedures for assessment, but not judicial interpretation, of cases involving AI and automated systems.” This approach respects and enables “the contextual approach to judicial interpretation practiced under EU non-discrimination law.”^160^ Our proposal here follows the same logic: SSOs should aim to set minimum testing and disclosure requirements in order to support procedurally consistent and well-informed local determinations of appropriate, case-specific ethical requirements for AI.

## 5 Conclusion

AI is a uniquely broad type of technology, capable in principle of augmenting or replacing human intellectual work in any sector. Despite this, the AIA seeks harmonised standards for abstract normative concepts which can apply to myriad applications and sectors. High-level standards describing a range of possible technologies and methods rather than specific, implementable ethical requirements are a highly probable outcome of the AIA standardisation process.^161^ Vague or abstract standards will require significant interpretation by providers, introducing additional questions around the political legitimacy of the answers they provide.

In this context, we suggest the best legitimate pathway forward is to pursue standards which ensure procedurally consistent and participatory local ethical decision-making. Standards developed under the AIA should adopt a position of ethical disclosure by default which exceeds the current reporting and participatory requirements of the AIA. Shifting discretion away from AI providers, local stakeholders should be empowered to request relevant documentation, including test results, impact assessments, transparency disclosures and explanations.

We have shown in this paper that the alternative pathways for standardisation addressing hard normative questions either lack legitimacy or are error prone. With the AIA’s horizontal approach to AI regulation, SSOs will expectably struggle to include all relevant stakeholders in the process. We thus cannot recommend SSOs answering hard normative questions themselves. This approach would likely also disregard the considerable normative uncertainty and principled disagreement around the proper use AI of in society. Furthermore, we cannot endorse SSOs tracking normative consensus. This is not merely because such consensus is currently lacking for many problematic issues around AI. As shown in this paper, the procedure of identifying consensus can be faulty. It could furthermore lead to SSOs extending the scope of private standardisation towards translating legal principles.

We therefore outlined an alternative approach, aiming to stop the ‘AI Ethics’ can from being kicked down the road and empower local ethical decision-makers with disclosure tools. Whether European SSOs will adopt ethical disclosure by default is an open question at the time of writing. Its answer will not least depend on the dynamics of the standardisation process and whose interests will prevail in shaping its outcome.^162^ Given the societal importance of regulating AI well, European SSOs should remain mindful of their limited political legitimacy but nevertheless be innovative enough to endow local stakeholders with the information and discretion necessary to provide implementable answers to hard normative questions around AI.

## Footnotes

1 For an overview of the history of the field, see: Melanie Mitchell, *Artificial Intelligence: A Guide for Thinking Humans* (Pelican 2020) 3–64.

2 Ryan Calo, ‘Artificial Intelligence Policy: A Primer and Roadmap’ (2017) 51 UC Davis Law Review 399, 402.

3 Will Douglas Heaven, ‘Hundreds of AI Tools Have Been Built to Catch Covid: None of Them Helped’ *MIT Technology Review* (Cambridge, MA, 30 July 2021) <https://www.technologyreview.com/2021/07/30/1030329/machine-learning-ai-failed-covid-hospital-diagnosis-pandemic/>.

4 Michael Roberts and others, ‘Common Pitfalls and Recommendations for Using Machine Learning to Detect and Prognosticate for COVID-19 Using Chest Radiographs and CT Scans’ (2021) 3 Nature Machine Intelligence 199; The Alan Turing Institute, ‘Data Science and AI in the Age of COVID-19: Reflections on the Response of the UK’s Data Science and AI Community to the COVID-19 Pandemic’ (2021); Laure Wynants and others, ‘Prediction Models for Diagnosis and Prognosis of Covid-19: Systematic Review and Critical Appraisal’ [2020] BMJ m1328.

5 Heaven (n 3).

6 Roberts and others (n 4) 203; The Alan Turing Institute (n 4) 12; Wynants and others (n 4) 7.

7 Roberts and others (n 4) 213.

8 The Alan Turing Institute (n 4) 12.

9 Regulation (EU) No 1025/2012 of the European Parliament and of the Council of 25 October 2012 on European standardisation, amending Council Directives 89/686/EEC and 93/15/EEC and Directives 94/9/EC, 94/25/EC, 95/16/EC, 97/23/EC, 98/34/EC, 2004/22/EC, 2007/23/EC, 2009/23/EC and 2009/105/EC of the European Parliament and of the Council and repealing Council Decision 87/95/EEC and Decision No 1673/2006/EC of the European Parliament and of the Council 2012 para (1).

10 On standards as a means of global governance, cf. Dieter Kerwer, ‘Rules That Many Use: Standards and Global Regulation’ (2005) 18 Governance 611, 616.

11 Martin Ebers, ‘Standardizing AI: The Case of the European Commission’s Proposal for an “Artificial Intelligence Act”*’ in Larry A DiMatteo, Cristina Poncibò and Michel Cannarsa (eds), *The Cambridge Handbook of Artificial Intelligence* (1st edn, Cambridge University Press 2022) 331.

12 On this, see, for example: Raymund Werle and Eric J Iversen, ‘Promoting Legitimacy in Technical Standardization’ (2006) 2 Science, Technology & Innovation Studies 19, 21–23.

13 Alan Beattie, ‘How the US, EU and China Compete to Set Industry Standards’ *Financial Times* (Brussels, 24 July 2019) <https://www.ft.com/content/0c91b884-92bb-11e9-aea1-2b1d33ac3271>.

14 European Commission, Proposal for a Regulation of the European Parliament and of the Council laying down harmonised rules on Artificial Intelligence (Artificial Intelligence Act) and amending certain Union legislative acts 2021 [COM(2021) 206 final]. At the time of writing, the Council of the European Union has published a compromise proposal for the AIA, while the Commission’s draft is still being negotiated in the European Parliament, cf. Council of the Europan Union, Proposal for a Regulation of the European Parliament and of the Council laying down harmonised rules on artificial intelligence (Artificial Intelligence Act) and amending certain Union legislative acts - General approach – Interinstitutional File: 2021/0106(COD) 2022 [14954/22].

15 AIA, page 3.

16 AIA, page 3.

17 Michelle Egan, *Constructing a European Market: Standards, Regulation, and Governance* (Oxford University Press 2001) 2.

18 Anu Bradford, *The Brussels Effect: How the European Union Rules the World* (1st edn, Oxford University Press 2020) <https://academic.oup.com/book/36491> accessed 12 January 2024.

19 Charlotte Siegmann and Markus Anderljung, ‘The Brussels Effect and Artificial Intelligence: How EU Regulation Will Impact the Global AI Market’ <https://arxiv.org/abs/2208.12645> accessed 12 January 2024; Alex Engler, ‘The EU AI Act Will Have Global Impact, but a Limited Brussels Effect’ (Brookings Institution 2022) <https://policycommons.net/artifacts/4141277/the-eu-ai-act-will-have-global-impact-but-a-limited-brussels-effect/4949540/> accessed 12 January 2024.

20 Michael Veale and Frederik Zuiderveen Borgesius, ‘Demystifying the Draft EU Artificial Intelligence Act — Analysing the Good, the Bad, and the Unclear Elements of the Proposed Approach’ (2021) 22 Computer Law Review International 97, 102.

21 Michael Veale and Frederik Zuiderveen Borgesius, ‘Demystifying the Draft EU Artificial Intelligence Act: Analysing the Good, the Bad, and the Unclear Elements of the Proposed Approach’ (2021) 22 Computer Law Review International 97, 104–105.

22 Jane Winn and Nicolas Jondet, ‘A “New Approach” to Standards and Consumer Protection’ (2008) 31 Journal of Consumer Policy 459, 463.

23 ibid.

24 Veale and Zuiderveen Borgesius (n 20) 104; Katerina Yordanova, ‘The EU AI Act: Balancing Human Rights and Innovation through Regulatory Sandboxes and Standardization’ (Competition Policy International 2022) 8; Kees Stuurman and Eric Lachaud, ‘Regulating AI. A Label to Complete the Proposed Act on Artificial Intelligence’ (2022) 44 Computer Law & Security Review 105657, 2; Natali Helberger and Nicholas Diakopoulos, ‘The European AI Act and How It Matters for Research into AI in Media and Journalism’ [2022] Digital Journalism 1, 5; Nathalie A Smuha and others, ‘How the EU Can Achieve Legally Trustworthy AI: A Response to the European Commission’s Proposal for an Artificial Intelligence Act’ [2021] SSRN Electronic Journal 54 <https://www.ssrn.com/abstract=3899991> accessed 26 October 2022; Christian Djeffal, ‘Democracy, AI Regulation and the Draft EU AI Act’ (*Transatlantic Policy Quarterly*, 4 March 2022) <http://turkishpolicy.com/article/1106/democracy-ai-regulation-and-the-draft-eu-ai-act>; Ebers (n 11) 332; The European Consumer Organisation (BEUC), ‘Regulating AI to Protect the Consumer: Position Paper on the AI Act’ (2021) BEUC-X-2021-088 25–27.

25 AIA, p. 13.

26 Veale and Zuiderveen Borgesius (n 20) 105.

27 European Commission, ‘Commission Implementing Decision of 22.5.2023 on a Standardisation Request to the European Committee for Standardisation and the European Committee for Electrotechnical Standardisation in Support of Union Policy on Artificial Intelligence’.

28 Cf. Graeme Auld and others, ‘Governing AI through Ethical Standards: Learning from the Experiences of Other Private Governance Initiatives’ (2022) 29 Journal of European Public Policy 1822; Hadrien Pouget, ‘The EU’s AI Act Is Barreling Toward AI Standards That Do Not Exist’ (*Lawfare*, 12 January 2023) <https://www.lawfareblog.com/eus-ai-act-barreling-toward-ai-standards-do-not-exist> accessed 17 February 2023; Hadrien Pouget and Johann Laux, ‘A Letter to the EU’s Future AI Office’ (3 October 2023) <https://carnegieendowment.org/2023/10/03/letter-to-eu-s-future-ai-office-pub-90683> accessed 25 January 2024; Manuel Wörsdörfer, ‘Mitigating the Adverse Effects of AI with the European Union’s Artificial Intelligence Act: Hype or Hope?’ (2024) 43 Global Business and Organizational Excellence 106; Manuel Wörsdörfer, ‘The E.U.’s Artificial Intelligence Act: An Ordoliberal Assessment’ [2023] AI and Ethics <https://link.springer.com/10.1007/s43681-023-00337-x> accessed 8 February 2024; Isabelle Hupont and others, ‘Documenting High-Risk AI: A European Regulatory Perspective’ (2023) 56 Computer 18; Laura Lucaj, Patrick Van Der Smagt and Djalel Benbouzid, ‘AI Regulation Is (Not) All You Need’, *2023 ACM Conference on Fairness, Accountability, and Transparency* (ACM 2023) <https://dl.acm.org/doi/10.1145/3593013.3594079> accessed 8 February 2024; Jonas Schuett, ‘Risk Management in the Artificial Intelligence Act’ [2023] European Journal of Risk Regulation 1; David Schneeberger and others, ‘The Tower of Babel in Explainable Artificial Intelligence (XAI)’ in Andreas Holzinger and others (eds), *Machine Learning and Knowledge Extraction*, vol 14065 (Springer Nature Switzerland 2023) <https://link.springer.com/10.1007/978-3-031-40837-3_5> accessed 8 February 2024.

29 In the context of AI standardisation alone, see: Yordanova (n 26) 7; Stuurman and Lachaud (n 26) 9; Smuha and others (n 26) 54; Djeffal (n 26); The European Consumer Organisation (BEUC) (n 26) 25–27; Ebers (n 11) 332–333; Veale and Zuiderveen Borgesius (n 20) 105–106. For a more general account, see the overview and references in: Jean-Christophe Graz and Christophe Hauert, ‘Translating Technical Diplomacy: The Participation of Civil Society Organisations in International Standardisation’ (2019) 33 Global Society 163, 167–168; Stefano Ponte, Peter Gibbon and Jakob Vestergaard (eds), *Governing through Standards: Origins, Drivers and Limitations* (Palgrave Macmillan 2011); Tim Büthe and Walter Mattli, *The New Global Rulers: The Privatization of Regulation in the World Economy* (Princeton University Press 2011); Egan (n 17).

30 Michelle Egan, ‘Regulatory Strategies, Delegation and European Market Integration’ (1998) 5 Journal of European Public Policy 485.

31 Büthe and Mattli (n 32). See also the recollection of the genesis of the ISO 26000 standard in section 3.1 and cf. Stephanie Bijlmakers and Geert van Calster, ‘You’d Be Surprised How Much It Costs to Look This Cheap! A Case Study of ISO 26000 on Social Responsibility’ in Panagiotis Delimatsis (ed), *The Law, Economics and Politics of International Standardisation* (1st edn, Cambridge University Press 2015) 277–284.

32 Sandra Wachter, Brent Mittelstadt and Chris Russell, ‘Why Fairness Cannot Be Automated: Bridging the Gap between EU Non-Discrimination Law and AI’ (2021) 41 Computer Law & Security Review 105567.

33 Brent Mittelstadt, ‘Principles Alone Cannot Guarantee Ethical AI’ (2019) 1 Nature Machine Intelligence 501.

34 Without claiming to be exhaustive, see the following list of contributions: Werle and Iversen (n 12); Linda Senden, ‘Towards a More Holistic Legitimacy Approach to Technical Standardisation in the EU’ in Mariolina Eliantonio and Caroline Cauffman, *The Legitimacy of Standardisation as a Regulatory Technique* (Edward Elgar Publishing 2020) <https://www.elgaronline.com/view/edcoll/9781789902945/9781789902945.00007.xml> accessed 31 October 2022; Robin Hoenkamp, George B Huitema and Adrienne JC de Moor-van Vugt, ‘The Neglected Consumer: The Case of the Smart Meter Rollout in the Netherlands’ (2011) 2 Renewable Energy Law and Policy Review 269; Caroline Cauffman and Marie Gérardy, ‘Competition Law as a Tool to Ensure the Legitimacy of Standard-Setting by European Standardisation Organisations?’ in Mariolina Eliantonio and Caroline Cauffman, *The Legitimacy of Standardisation as a Regulatory Technique* (Edward Elgar Publishing 2020); Egan (n 33); Egan (n 17); Harm Schepel, *The Constitution of Private Governance: Product Standards in the Regulation of Integrating Markets* (Hart Pub 2005); Büthe and Mattli (n 32); Graz and Hauert (n 32).

35 On input and output legitimacy in this context, see: Werle and Iversen (n 12) 27.

36 On throughput legitimacy in this context, see: Senden (n 38).

37 Cf. Regulation 1025/2012, recital (1).

38 On the presumption of conformity, cf. Regulation 1025/2012, recital (5).

39 Mittelstadt (n 37) 501–507.

40 Thomas Metzinger, ‘Ethics Washing Made in Europe’ *Der Tagesspiegel* (Berlin, 8 April 2019) <https://www.tagesspiegel.de/politik/ethics-washing-made-in-europe-5937028.html>.

41 See the references in: Wachter, Mittelstadt and Russell (n 36) 2.

42 Shira Mitchell and others, ‘Algorithmic Fairness: Choices, Assumptions, and Definitions’ (2021) 8 Annual Review of Statistics and Its Application 141.

43 Wachter, Mittelstadt and Russell (n 36).

44 Identifying these four challenges: Mittelstadt (n 37).

45 WB Gallie, ‘Essentially Contested Concepts’ (1955) 56 Proceedings of the Aristotelian Society 167; Mittelstadt (n 37).

46 A search on the CEN/CENELEC database (https://standards.cencenelec.eu/dyn/www/f?p=CEN:105::RESET) on 20 February 2023 with the search keyword “ethic” produced 20 results.

47 See most recently: International Organization for Standardization (ISO) and International Electrotechnical Commission (IEC), ‘Information Technology — Artificial Intelligence — Management System’ (2023) ISO/IEC 42001:2023(en). See also: International Organization for Standardization (ISO) and International Electrotechnical Commission (IEC), ‘Information Technology — Artificial Intelligence (AI) — Bias in AI Systems and AI Aided Decision Making’ (2021) ISO/IEC TR 24027:2021(E) v.

48 European Commission. Joint Research Centre., *AI Watch: Artificial Intelligence Standardisation Landscape Update*. (Publications Office 2023) 6 <https://data.europa.eu/doi/10.2760/131984> accessed 16 February 2023.

49 Yordanova (n 26) 8; Bijlmakers and van Calster (n 34) 287.

50 Bijlmakers and van Calster (n 34) 275.

51 International Organization for Standardization (ISO) and International Electrotechnical Commission (IEC), ‘Information Technology — Artificial Intelligence (AI) — Bias in AI Systems and AI Aided Decision Making’ (n 51) v.

52 Bijlmakers and van Calster (n 34) 275–278. On MSS, see further: Iñaki Heras-Saizarbitoria and Olivier Boiral, ‘ISO 9001 and ISO 14001: Towards a Research Agenda on Management System Standards*: Research Agenda on Management System Standards’ (2013) 15 International Journal of Management Reviews 47.

53 Bijlmakers and van Calster (n 34) 281.

54 ibid.

55 ISO Advisory Group on Social Responsibility, ‘Recommendations to the ISO Technical Management Board’ (2004) ISO/TMB AG CSR N32 1; Bijlmakers and van Calster (n 34) 280.

56 ISO Advisory Group on Social Responsibility (n 59) 1.

57 ibid.

58 Bijlmakers and van Calster (n 34) 284.

59 International Organization for Standardization (ISO), ‘Discovering ISO 26000’ (2018) 8 <https://www.iso.org/files/live/sites/isoorg/files/store/en/PUB100258.pdf>.

60 Pavel Castka and Michaela A Balzarova, ‘The Impact of ISO 9000 and ISO 14000 on Standardisation of Social Responsibility—an inside Perspective’ (2008) 113 International Journal of Production Economics 74, 84–85; Bijlmakers and van Calster (n 34) 281.

61 Bijlmakers and van Calster (n 34) 281.

62 Rüdiger Hahn, ‘Standardizing Social Responsibility? New Perspectives on Guidance Documents and Management System Standards for Sustainable Development’ (2012) 59 IEEE Transactions on Engineering Management 717, 725.

63 Bijlmakers and van Calster (n 34) 282.

64 ISO Advisory Group on Social Responsibility (n 59) 1.

65 Bijlmakers and van Calster (n 34) 282.

66 ISO Advisory Group on Social Responsibility (n 59) 1.

67 Bijlmakers and van Calster (n 34) 282.

68 ibid 283–284; ISO Advisory Group on Social Responsibility (n 59) 1.

69 Halina Ward, *ISO 26000 and Global Governance for Sustainable Development* (International Institute for Environment and Development 2012) 33; Bijlmakers and van Calster (n 34) 297.

70 Ward (n 73) 35–37.

71 Bijlmakers and van Calster (n 34) 297–298; Ward (n 73) 26–37.

72 Bijlmakers and van Calster (n 34) 298.

73 International Organization for Standardization (ISO), ‘Enabling an AI-Ready Culture’ (22 November 2021) <https://www.iso.org/news/ref2763.html>.

74 ibid.

75 ibid.

76 ibid.

77 ibid.

78 International Organization for Standardization (ISO) and International Electrotechnical Commission (IEC), ‘Information Technology — Artificial Intelligence — Management System’ (n 51).

79 ibid.

80 Hahn (n 66) 718–719.

81 Cf. https://www.iso.org/committee/6794475/x/catalogue/p/1/u/0/w/0/d/0, last accessed on 20 February 2023.

82 International Organization for Standardization (ISO) and International Electrotechnical Commission (IEC), ‘Information Technology — Artificial Intelligence — Overview of Trustworthiness in Artificial Intelligence’ (2020) ISO/IEC TR 24028:2020 (E).

83 ibid 1.

84 ibid 8.

85 ibid.

86 Johann Laux, Sandra Wachter and Brent Mittelstadt, ‘Trustworthy Artificial Intelligence and the European Union AI Act: On the Conflation of Trustworthiness and Acceptability of Risk’ [2023] Regulation & Governance rego.12512.

87 International Organization for Standardization (ISO) and International Electrotechnical Commission (IEC), ‘Information Technology — Artificial Intelligence — Overview of Trustworthiness in Artificial Intelligence’ (n 89) 16–17.

88 ibid 32.

89 ibid.

90 ibid 13.

91 ibid 13.

92 ibid 23.

93 International Organization for Standardization (ISO) and International Electrotechnical Commission (IEC), ‘Information Technology — Artificial Intelligence (AI) — Bias in AI Systems and AI Aided Decision Making’ (n 51).

94 ibid vi.

95 ibid 1.

96 ibid 3–6.

97 ibid 5.

98 ibid 6–27.

99 ibid 18.

100 ibid.

101 International Organization for Standardization (ISO), ‘Enabling an AI-Ready Culture’ (n 77).

102 ibid.

103 International Organization for Standardization (ISO) and International Electrotechnical Commission (IEC), ‘Information Technology — Artificial Intelligence — Overview of Ethical and Societal Concerns’ (2022) ISO/IEC TR 24368:2022.

104 ibid vi.

105 ibid 1.

106 ibid 6.

107 ibid 4.

108 ibid 7.

109 ibid.

110 ibid.

111 ibid 8.

112 ibid.

113 ibid 10.

114 ibid.

115 International Organization for Standardization (ISO) and International Electrotechnical Commission (IEC), ‘Information Technology — Governance of IT — Governance Implications of the Use of Artificial Intelligence by Organizations’ (2022) ISO/IEC 38507.

116 ibid 6.

117 ibid 10.

118 ibid 26.

119 ibid 10.

120 ibid 15.

121 International Organization for Standardization (ISO) and International Electrotechnical Commission (IEC), ‘Information Technology — Artificial Intelligence — Management System’ (n 51) vi.

122 International Organization for Standardization (ISO), ‘Management System Standards’ <https://www.iso.org/management-system-standards.html> accessed 30 January 2024.

123 International Organization for Standardization (ISO) and International Electrotechnical Commission (IEC), ‘Information Technology — Artificial Intelligence — Management System’ (n 51) 1.

124 ibid vi.

125 See, for example, ibid 6, 29.

126 International Organization for Standardization (ISO) and International Electrotechnical Commission (IEC), ‘Information Technology — Artificial Intelligence — Management System’ (n 51) 9.

127 ibid.

128 ibid 42 (B.9.3). On the difficulty of implementing “meaningful” human oversight: Ben Green, ‘The Flaws of Policies Requiring Human Oversight of Government Algorithms’ (2022) 45 Computer Law & Security Review 105681; Johann Laux, ‘Institutionalised Distrust and Human Oversight of Artificial Intelligence: Towards a Democratic Design of AI Governance under the European Union AI Act’ [2023] AI & SOCIETY <https://doi.org/10.1007/s00146-023-01777-z> accessed 24 October 2023.

129 International Organization for Standardization (ISO) and International Electrotechnical Commission (IEC), ‘Information Technology — Artificial Intelligence — Management System’ (n 51) 5, 21, 27, 28.

130 ibid 19 (Annex A, A.7.4).

131 ibid 25–6.

132 ibid 25 (B.4.3); 38 (B.7.3).

133 Tim Rädsch and others, ‘Labelling Instructions Matter in Biomedical Image Analysis’ (2023) 5 Nature Machine Intelligence 273; Johann Laux, Fabian Stephany and Alice Liefgreen, ‘The Economics of Human Oversight: How Norms and Incentives Affect Costs and Performance of AI Workers’ [2023] SSRN Electronic Journal <https://www.ssrn.com/abstract=4673217> accessed 29 January 2024.

134 International Organization for Standardization (ISO), ‘Standards by ISO/IEC JTC 1/SC 42’ <https://www.iso.org/committee/6794475/x/catalogue/p/0/u/1/w/0/d/0> accessed 29 January 2024.

135 For ISO, see: International Organization for Standardization (ISO) and International Electrotechnical Commission (IEC), ‘ISO/IEC Directives, Part 2: Principles and Rules for the Structure and Drafting of ISO and IEC Documents’ (2021) ninth edition 3.1 <https://www.iso.org/sites/directives/current/part2/index.xhtml#_idTextAnchor009>. For CEN/CENELEC, see: European Committee for Standardization (CEN) and European Committee for Electrotechnical Standardization (CENELEC), ‘Types of Deliverables’ (2022) <https://www.cencenelec.eu/european-standardization/european-standards/types-of-deliverables/>.

136 Hahn (n 66) 718.

137 European Committee for Standardization (CEN), ‘CEN Deliverables’ (24 March 2016) <https://boss.cen.eu/reference-material/guidancedoc/pages/del/>.

138 ibid.

139 ibid.

140 Gallie (n 49); Mittelstadt (n 37).

141 European Commission, ‘Commission Launches AI Innovation Package to Support Artificial Intelligence Startups and SMEs’ (24 January 2024) <https://ec.europa.eu/commission/presscorner/detail/en/ip_24_383> accessed 25 January 2024.

142 Pouget and Laux (n 31).

143 On these normative questions of institutional design, see: Johann Laux, *Public Epistemic Authority: Normative Institutional Design for EU Law* (Mohr Siebeck 2022). For an updated version of the argument, see: Johann Laux, ‘Public Epistemic Authority: An Epistemic Framework for the Institutional Legitimacy of International Adjudication’ [2019] Jean Monnet Working Paper 5/19 <https://jeanmonnetprogram.org/wp-content/uploads/JMWP-05-Johann-Laux.pdf>.

144 On this issue of blaming frontline workers for AI’s errors, see: Ben Green, ‘The Flaws of Policies Requiring Human Oversight of Government Algorithms’ (2022) 45 Computer Law & Security Review 105681, 10–11.

145 ibid 10.

146 Mittelstadt (n 37); Anna Jobin, Marcello Ienca and Effy Vayena, ‘The Global Landscape of AI Ethics Guidelines’ (2019) 1 Nature Machine Intelligence 389.

147 Ebers (n 11).

148 ISO Advisory Group on Social Responsibility (n 59) 1.

149 Brent Mittelstadt, Chris Russell and Sandra Wachter, ‘Explaining Explanations in AI’, *Proceedings of the Conference on Fairness, Accountability, and Transparency* (Association for Computing Machinery 2019) <https://doi.org/10.1145/3287560.3287574> accessed 27 July 2020; Brent Mittelstadt, ‘Interpretability and Transparency in Artificial Intelligence’ in Carissa Véliz (ed), *The Oxford Handbook of Digital Ethics* (Oxford University Press 2022) <https://doi.org/10.1093/oxfordhb/9780198857815.013.5> accessed 13 September 2022; Tim Miller, ‘Explanation in Artificial Intelligence: Insights from the Social Sciences’ (2019) 267 Artificial Intelligence 1.

150 Dino Pedreshi, Salvatore Ruggieri and Franco Turini, ‘Discrimination-Aware Data Mining’, *Proceedings of the 14th ACM SIGKDD International Conference on Knowledge Discovery and Data Mining* (ACM 2008) <http://doi.acm.org/10.1145/1401890.1401959> accessed 27 September 2017; Rachel KE Bellamy and others, ‘AI Fairness 360: An Extensible Toolkit for Detecting, Understanding, and Mitigating Unwanted Algorithmic Bias’ [2018] arXiv:1810.01943 [cs] <http://arxiv.org/abs/1810.01943> accessed 15 March 2019; Salvatore Ruggieri, Dino Pedreschi and Franco Turini, ‘Data Mining for Discrimination Discovery’ (2010) 4 ACM Transactions on Knowledge Discovery from Data (TKDD) 9.

151 Sahil Verma and Julia Rubin, ‘Fairness Definitions Explained’, *2018 IEEE/ACM International Workshop on Software Fairness (FairWare)* (IEEE 2018); Moritz Hardt, Eric Price and Nati Srebro, ‘Equality of Opportunity in Supervised Learning’, *Advances in Neural Information Processing Systems* (2016); Cynthia Dwork and others, ‘Fairness Through Awareness’ [2011] arXiv:1104.3913 [cs] <http://arxiv.org/abs/1104.3913> accessed 15 February 2016; Matt J Kusner and others, ‘Counterfactual Fairness’ in I Guyon and others (eds), *Advances in Neural Information Processing Systems 30* (Curran Associates, Inc 2017) <http://papers.nips.cc/paper/6995-counterfactual-fairness.pdf> accessed 17 July 2019; Bellamy and others (n 158).

152 Christoph Molnar, *Interpretable Machine Learning* (2020) <https://christophm.github.io/interpretable-ml-book/> accessed 31 January 2019; Miller (n 157); Mittelstadt (n 157).

153 Timnit Gebru and others, ‘Datasheets for Datasets’ <https://arxiv.org/abs/1803.09010> accessed 1 October 2018; Margaret Mitchell and others, ‘Model Cards for Model Reporting’ [2019] Proceedings of the Conference on Fairness, Accountability, and Transparency - FAT* ‘19 220; Sarah Holland and others, ‘The Dataset Nutrition Label: A Framework To Drive Higher Data Quality Standards’ [2018] arXiv:1805.03677 [cs] <http://arxiv.org/abs/1805.03677> accessed 1 October 2018.

154 Dillon Reisman and others, ‘Algorithmic Impact Assessments: A Practical Framework for Public Agency Accountability’ [2018] AI Now Institute 1; L Edwards, D McAuley and L Diver, ‘From Privacy Impact Assessment to Social Impact Assessment’, *2016 IEEE Security and Privacy Workshops (SPW)* (2016).

155 In this regard, the cooperation between UNESCO’s Social and Human Sciences Sector, the European Commission, and the Dutch Authority for Digital Infrastructure on “Supervising AI by Competent Authorities” presents an opportunity to develop an ethical disclosure approach, cf. Ministerie van Economische Zaken en Klimaat, ‘UNESCO-EC-Netherlands AI Supervision Collaboration’ <https://www.rdi.nl/onderwerpen/kunstmatige-intelligentie/nieuws/2023/10/05/unesco-ec-netherlands-ai-supervision-collaboration> accessed 8 February 2024.

156 SSOs and the AIA describe the need for participatory standardisation by encouraging involvement from civil society stakeholders in the standards setting process. Our proposal likewise calls for a participatory element, but in a different way. Participation should extend to implementation and enforcement of standards.

157 Bijlmakers and van Calster (n 34) 283–284; ISO Advisory Group on Social Responsibility (n 59) 1.

158 L Bergkamp, ‘Research Ethics Committees and the Regulation of Medical Experimentation with Human Beings in the Netherlands’ (1988) 7 Medicine and Law 65; Annette Markham and others, ‘Ethical Decision-Making and Internet Research: Version 2.0’ [2012] Association of Internet Researchers <http://www.uwstout.edu/ethicscenter/upload/aoirethicsprintablecopy.pdf> accessed 2 January 2017; Wachter, Mittelstadt and Russell (n 36).

159 Wachter, Mittelstadt and Russell (n 36).

160 ibid 4.

161 Ebers (n 11).

162 For an analysis of the possible pathways for *these* procedures, cf. Auld and others (n 31).
